# Effect of fire smoke on some biochemical parameters in firefighters of Saudi Arabia

**DOI:** 10.1186/1745-6673-3-33

**Published:** 2008-12-11

**Authors:** Abdulrahman L Al-Malki, Ameen M Rezq, Mohamed H Al-Saedy

**Affiliations:** 1 Biochemistry Department, Faculty of Science, King Abdualziz University, Jeddah, KSA; 2 Medical Biochemistry Department, Faculty of Medicine, Cairo University, Cairo, Egypt; 3 Civil Defense forces, Madinah, KSA

## Abstract

**Background:**

Firefighters who are facing fires, are frequently exposed to hazardous materials including carbon monoxide, hydrogen cyanide, hydrogen chloride, benzene, sulphur dioxide, etc. This study aimed to evaluate some relevant serum biochemical and blood hematological changes in activity involved firefighters in comparison to normal subjects.

**Subjects and Methods:**

Two groups of male firefighters volunteered to participate in the study. The first included 28 firefighters from Jeddah, while the second included 21 firefighters from Yanbu, with overall age ranged 20–48 years. An additional group of 23 male non-firefighters volunteered from both cities as normal control subjects, of age range 20–43 years. Blood samples were collected from all volunteer subjects and investigated for some relevant serum biochemical and blood hematological changes.

**Results:**

The results obtained showed that, there were statistically significant differences in liver function, kidney function, serum lipid profile, cortisol, creatine kinase, lactate dehydrogenase, iron and its biologically active derivatives, and blood picture in firefighters as compared with the normal control group. These results indicate that, fire smoke mainly affects serum biochemical and blood hematological parameters. Such results might point out to the need for more health protective and prophylactic measures to avoid such hazardous health effects that might endanger firefighters under their highly drastic working conditions.

**Conclusion:**

Besides using of personal protective equipments for firefighters to protect them against exposure to toxic materials of fire smoke, it is recommended that, firefighters must be under continuous medical follow up through a standard timetabled medical laboratory investigations to allow for early detection of any serum biochemical or blood hematological changes that might happen during their active service life and to allow for early treatment whenever necessary.

## Background

Fire Smoke is actually produced by two chemical processes: Combustion, (oxidation) and pyrolysis, [1]. Oxidation is the process by which oxygen chemically combines with combustible molecules and degrades them to smaller compounds. Heat and light are generated as byproducts. Pyrolysis is purely a function of heat and refers to the direct liberation of combustible materials constituents through melting and boiling. Sufficient heat may lead to the thermal breakdown of larger to smaller molecules, some of which may be highly toxic. The individual products of oxidation and pyrolysis may also react and thereby produce hundreds or thousands of toxic gaseous compounds [2]. The most common toxic gases in fire smoke are carbon monoxide and carbon dioxide. Other gases may also be produced in toxicologically significant quantities, depending on the chemical structure of the burning material and the fire conditions [3]. Carbon monoxide and hydrogen cyanide as narcotic gases are principally implicated in the death of fire victims [4]. Hydrogen cyanide poisoning is synergistic with that of carbon monoxide, and exposure may be more common as parent compounds such as polyurethane, acrylonitrile, and nylon [5].

Many of the above mentioned materials have been implicated in the production of cardiovascular, respiratory or neoplastic diseases, which may provide an explanation for the alleged increased risk for these illnesses among firefighters [6]. Most fatalities from fires are not due to burns, but are a result of inhalation of toxic gases produced during combustion [7]. The third major cause of death is the intense sensory irritations of the smoke that lead rapidly to functional impairment [8].

The main objective of this research is to study the effect of fire smoke on firefighters of Jeddah and Yanbu cities by evaluation of the serum biochemical and blood hematological changes in those firefighters and compare them with normal control subjects.

## Subjects and Methods

The study protocol approved by the local ethics committee. A written informed consent were obtained from all subjects. Two groups of male firefighters volunteered to participate in the study: The first included 28 firefighters from Jeddah, age ranged (20–45). The second included 21 firefighters from Yanbu, age ranged (20–48). An additional group of 23 male non-firefighters volunteered from both cities as normal control subjects, age ranged, (20–43). All subjects were clinically investigated to exclude those who were suffering from acute and chronic illnesses (as diabetic, hypertension and cardiac diseases. In particular, normal chest x-ray was an essential inclusion clinical parameter for the normal control groups. All firefighters volunteers were randomly chosen for participation. All participants were informed well with the objective and the course of the study.

Ten milliliters of venous blood were withdrawn from each participant of the two firefighters groups within the first hour after firefighting of a fire accident regardless of time, scale nor type of the fire accidents they faced, without anticoagulant for subsequent separation of serum and measurement of the required biochemical parameters.

### Serum Biochemical Analysis

Dade Behring, (Dimention^® ^Xpand^®^, Clinical Chemistry System) has been used for measurement of all biochemical parameters except otherwise specified ones. This instrument is based on integrated multisensory technology, (IMT) and manufactured by Dade Behring Inc, USA.

The Cell- dyn^® ^1800 Hematology Analyzer was used to perform a complete blood count, (CBC), Platelet Count and a Three Part Differential. It is based on the proven technology and manufactured by Abbott Diagnostics, Abbott Laboratories, 2000 Abbott Park Road, Abbott Park, IL 60064, USA[9].

### Statistical Analysis

Statistical analysis was performed on a PC using SPSS, V.13, (special package for social sciences). Data are presented as arithmetic mean ± S.D., with subsequent use of z-test for the determination of significance of difference between two proportions. Student *t *test was used for the determination of the significance of difference between sample means.

## Results

From table, (1) it is evident that serum urea nitrogen, low density lipoprotein, (LDL-C), creatine kinase, (CK) and lactate dehydrogenase, (LDH) were statistically significantly elevated in Jeddah firefighters as compared to normal control group, (p < 0.001; p < 0.01; p < 0.005 and p < 0.005 respectively), while non-significant changes were observed in all other studied parameters as compared to normal control group.

**Table 1:** Statistical Analysis of Liver Function and Kidney Function Tests, serum lipid profile and other biochemical parameters in Jeddah Firefighters as Compared to the Normal Control Group, (mean ± S.D.)

	**Parameters**	**Normal Control Group**	**n**	Jeddah FFs*	**n**	**t-test**	**p- value**
**Liver Function tests**	**ALP (u/l)**	90.15 ± 23.23	13	86.25 ± 27.59	28	0.4418	N.S.

	**ALT (u/l)**	50.26 ± 16.60	23	54.07 ± 25.52	28	0.6164	N.S.

	**AST (u/l)**	26.45 ± 12.12	11	29.19 ± 8.70	27	0.781341	N.S.

	**GGT (u/l)**	39.56 ± 14.38	9	44.54 ± 14.99	28	0.874940	N.S

	**Total Bilirubin (mg/dl)**	0.58 ± 0.21	4	0.55 ± 0.13	21	0.3267	N.S.

	**Direct Bilirubin (mg/dl)**	0.15 ± 0.13	4	0.16 ± 0.09	21	0.0964	N.S.

	**Total Protein (g/dl)**	7.44 ± 0.58	5	7.58 ± 0.84	25	0.3645	N.S.

	**Urea nitrogen (mmol/l)**	3.74 ± 0.97	10	5.13 ± 1.05	28	3.6375	p < 0.001

	**Albumin (g/dl)**	4.08 ± 0.40	6	4.38 ± 0.65	26	1.0754	N.S.

**Kidney Function tests**	**Uric Acid (mg/dl)**	6.32 ± 1.24	8	5.89 ± 0.97	24	1.0032	N.S.

	**Creatinine (umol/l)**	85.69 ± 12.76	23	86.02 ± 22.86	28	0.0619	N.S.

	**sodium (mmol/l)**	139.78 ± 3.40	23	141.29 ± 2.52	28	1.812	N.S.

	**potassium (mmol/l)**	4.31 ± 0.40	23	4.37 ± 0.6121	27	0.4385	N.S.

	**Calcium(mg/dl)**	9.32 ± 0.63	5	9.25 ± 0.440	28	0.290	N.S.

	**Chloride (mmol/l)**	102.57 ± 2.62	14	101.46 ± 1.64	28	1.679	N.S.

	**Phosphorous (mmol/l)**	1.10 ± 0.13	8	----	---	----	----

**Lipid Profile**	**Total Cholesterol (mmol/l)**	4.67 ± 0.66	23	4.96 ± 0.86	28	1.3250	N.S.

	**HDL-C (mg/dl)**	40.60 ± 5.92	11	44.29 ± 7.62	28	1.4393	N.S.

	**LDL-C (mg/dl)**	106.70 ± 16.72	11	137.37 ± 34.022	27	2.8368	p < 0.01

	**Triglyceride (mg/dl)**	125.33 ± 61.25	23	125.33 ± 61.25	25	0.8053	N.S.

**Others**	**Glucose (mmol/l)**	5.78 ± 1.73	23	5.64 ± 1.72	28	0.2789	N.S.

	**Cortisol (nmol/l)**	398.76 ± 136.28	21	380.70 ± 114.06	25	0.489502	N.S.

	**CK (u/l)**	112.95 ± 33.47	22	183.54 ± 93.73	28	3.36134	p < 0.005

	**LDH (u/l)**	143.17 ± 21.63	18	241.82 ± 124.40	27	3.31891	p < 0.005

Table, (2) shows that serum alanine transaminase, (ALT), direct bilirubin, (DBIL), serum urea nitrogen, albumin, creatine kinase, (CK) and lactate dehydrogenase, (LDH) were statistically significantly elevated, (p < 0.01; p < 0.005; p < 0.05; p < 0.05; p < 0.05 and p < 0.05 respectively), while serum chloride and cortisol level were statistically significantly decreased, (p < 0.005 and p < 0.05 respectively) in Yanbu firefighters as compared to normal control group, but there were non-significant changes in all other parameters as compared to normal control group.

**Table 2:** Statistical Analysis of Liver Function and Kidney Function Tests, serum lipid profile and other biochemical parameters in Yanbu Firefighters as Compared to the Normal Control Group, (mean ± S.D.).

	**Parameters**	**Normal Control Group**	**n**	Yanbu FFs*	**n**	**t-test**	**p- value**
**Liver Function tests**	**ALP (u/l)**	90.15 ± 23.23	13	87.45 ± 16.18	11	0.3241	N.S.

	**ALT (u/l)**	50.26 ± 16.60	23	68.27 ± 23.17	15	2.7939	p < 0.01

	**AST (u/l)**	26.45 ± 12.12	11	23.07 ± 8.45	14	0.8224	N.S.

	**GGT (u/l)**	39.56 ± 14.38	9	54.89 ± 19.66	9	1.88837	N.S.

	**Total Bilirubin (mg/dl)**	0.58 ± 0.21	4	0.69 ± 0.27	10	0.7613	N.S.

	**Direct Bilirubin (mg/dl)**	0.15 ± 0.13	4	0.32 ± 0.02	10	4.2320	p < 0.005

	**Total Protein (g/dl)**	7.44 ± 0.58	5	7.45 ± 0.35	10	0.0422	N.S.

	**Urea nitrogen (mmol/l)**	3.74 ± 0.97	10	4.80 ± 1.25	20	2.3336	p < 0.05

	**Albumin (g/dl)**	4.08 ± 0.40	6	4.48 ± 0.27	10	2.4082	p < 0.05

**Kidney Function tests**	**Uric Acid (mg/dl)**	6.32 ± 1.24	8	5.90 ± 0.67	3	0.5553	N.S.

	**Creatinine (umol/l)**	85.69 ± 12.76	23	93.45 ± 19.57	21	1.5728	N.S.

	**sodium (mmol/l)**	139.78 ± 3.40	23	139.19 ± 4.00	21	0.531	N.S,

	**potassium (mmol/l)**	4.31 ± 0.40	23	4.22 ± 0.53	21	0.638	N.S.

	**Calcium(mg/dl)**	9.32 ± 0.63	5	9.63 ± 0.25	4	0.903	N.S.

	**Chloride (mmol/l)**	102.57 ± 2.62	14	99.60 ± 2.70	20	1.193	p < 0.005

	**Phosphorous (mmol/l)**	1.10 ± 0.13	8	1.08 ± 0.25	9	0.226	N.S,

**Lipid Profile**	**Total Cholesterol (mmol/l)**	4.67 ± 0.66	23	5.05 ± 0.98	21	1.5100	N.S.

	**HDL-C (mg/dl)**	40.60 ± 5.92	11	42.10 ± 6.23	20	0.6531	N.S.

	**LDL-C (mg/dl)**	106.70 ± 16.72	11	122.53 ± 31.95	14	1.4867	N.S.

	**Triglyceride (mg/dl)**	125.33 ± 61.25	23	168.29 ± 109.47	19	1.60489	N.S.

**Others**	**Glucose (mmol/l)**	5.78 ± 1.73	23	5.50 ± 1.06	21	0.6457	N.S.

	**Cortisol (nmol/l)**	398.76 ± 136.28	21	307.55 ± 140.03	19	2.08629	p < 0.05

	**CK (u/l)**	112.95 ± 33.47	22	158.00 ± 85.53	19	2.28012	p < 0.05

	**LDH (u/l)**	143.17 ± 21.63	18	164.20 ± 28.21	20	2.55740	p < 0.05

(* firefighters)

On comparison between Jeddah firefighters and Yanbu firefighters, it is evident from table, (3) that serum aspartate transaminase, (AST), lactate dehydrogenase, (LDH), sodium and chloride were statistically significantly elevated, (p < 0.05; p < 0.01; p < 0.05 and p < 0.005 respectively), while serum direct bilirubin, (DBIL) was statistically significantly decreased, (p < 0.0001) in Jeddah firefighters as compared to Yanbu firefighters. However non-significant changes in other studied parameters were observed in Jeddah firefighters as compared to Yanbu firefighters.

**Table 3:** Statistical Analysis of liver function and kidney function tests, serum lipid Profile and Other Biochemical Parameters in Yanbu Firefighters as Compared to Jeddah Firefighters, (mean ± S.D.).

	**Parameters**	Jeddah FFs*	**n**	Yanbu FFs*	**n**	**t-test**	**p- value**
**Liver Function tests**	**ALP (u/l)**	86.25 ± 27.59	28	87.45 ± 16.18	11	0.1353	N.S.

	**ALT (u/l)**	54.07 ± 25.52	28	68.27 ± 23.17	15	1.7930	N.S.

	**AST (u/l)**	29.19 ± 8.70	27	23.07 ± 8.45	14	2.1539	p < 0.05

	**GGT (u/l)**	44.54 ± 14.99	28	54.89 ± 19.66	9	1.6700	N.S.

	**Total Bilirubin (mg/dl)**	0.55 ± 0.13	21	0.69 ± 0.27	10	1.9849	N.S.

	**Direct Bilirubin (mg/dl)**	0.16 ± 0.09	21	0.32 ± 0.02	10	5.3930	p < 0.0001

	**Total Protein (g/dl)**	7.58 ± 0.84	25	7.45 ± 0.35	10	0.4857	N.S.

	**Urea nitrogen (mmol/l)**	5.13 ± 1.05	28	4.80 ± 1.25	20	0.9902	N.S.

	**Albumin (g/dl)**	4.38 ± 0.65	26	4.48 ± 0.27	10	0.4681	N.S.

**Kidney Function tests**	**Uric Acid (mg/dl)**	5.89 ± 0.97	24	5.90 ± 0.67	3	0.0029	N.S.

	**Creatinine (umol/l)**	86.02 ± 22.86	28	93.45 ± 19.57	21	1.1967	N.S.

	**sodium (mmol/l)**	141.29 ± 2.52	28	139.19 ± 4.00	21	2.246	p < 0.05

	**potassium (mmol/l)**	4.37 ± 0.612	27	4.22 ± 0.53	21	0.64	N.S.

	**Calcium(mg/dl)**	9.25 ± 0.440	28	9.63 ± 0.25	4	1.626	N.S.

	**Chloride (mmol/l)**	101.46 ± 1.642	28	99.60 ± 2.70	20	2.968	p < 0.005

	**Phosphorous (mmol/l)**	----	---	1.08 ± 0.25	9	----	----

**Lipid Profile**	**Total Cholesterol (mmol/l)**	4.96 ± 0.86	28	5.05 ± 0.98	21	0.3288	N.S.

	**HDL-C (mg/dl)**	44.29 ± 7.62	28	42.10 ± 6.23	20	1.0548	N.S.

	**LDL-C (mg/dl)**	137.37 ± 34.022	27	122.53 ± 31.95	14	1.3512	N.S.

	**Triglyceride (mg/dl)**	125.33 ± 61.25	25	168.29 ± 109.47	19	1.6048	N.S.

**Others**	**Glucose (mmol/l)**	5.64 ± 1.72	28	5.50 ± 1.06	21	0.3469	N.S.

	**Cortisol (nmol/l)**	380.70 ± 114.06	25	307.55 ± 140.03	19	1.909642	N.S.

	**CK (u/l)**	183.54 ± 93.73	28	158.00 ± 85.53	19	0.9489	N.S.

	**LDH (u/l)**	241.82 ± 124.40	27	164.20 ± 28.21	20	2.7315	p < 0.01

(* firefighters)

## Discussion

Many of the substances identified in fire smoke are suspected human carcinogens or co-carcinogens. These compounds include many polycyclic aromatic hydrocarbons, (PAHs) which are almost formed from all types of combustion. The carcinogenicity of PAHs is associated with their subsequent covalent binding to critical targets in DNA[10]. Mutagens are toxic agents that cause genetic changes to the genetic material, (DNA) such that changes will propagate through generations. e.g. formaldehyde, acrolein, ethylene oxide, hydrogen peroxide and benzene[11].

All body organs and tissues could be affected by sutch toxic compounds. As liver cells are damaged, ALT leaks into the bloodstream leading to a rise in the serum levels. Any form of hepatic cell damage can result in an elevation in ALT[12]. In the present study, statistically significant increase, (p < 0.01) in the level of ALT has been found in Yanbu firefighters as compared to normal controls, (table 1) indicate of hepatic cell affection.

Concerning aspartate transaminase, (AST). it is raised in acute liver damage, but is also present in red cells, cardiac and skeletal muscle and is therefore not specific to the liver. The ratio of AST to ALT is sometimes useful in differentiating between causes of liver damage. AST levels are raised in shock and after excersise[13]. In table, (1) it is shown that there is a statistically significant increase, (p < 0.05) in serum AST in Jeddah firefighters over yanbu firefighters which might point out to the difference in the types of fires they fight.

Another enzyme, gamma glutamyl transpeptidase, (GGT) an indicator of early liver cell damage or cholestatic disease. Serum level of GGT is commonly elevated in patients with acute hepatitis although the rise in GGT is usually less than that of the transaminases. Serum GGT may also be elevated in response to many toxins. Myocardial infarction, cardiac failure, diabetes and pancreatitis can also increase serum GGT[14]. The present work showed statistically non-significant differences in serum GGT among the studied groups, (tables 1, 2 and 3).

Apart from enzymes, total bilirubin level is elevated in various forms of liver disease such as cirrhosis, hepatitis and obstructions of the hepatobiliary system such as gallstones or tumors. Elevated total bilirubin level is also observed in cases of intravascular hemolysis[15]. The results at the present study, no statistically significant differences between the studied groups. However as direct bilirubin which is formed only by the liver, and therefore, it is specific for hepatic or biliary disease as in obstructive liver diseases. Yanbu firefighters showed statistically significant increase in direct bilirubin over the normal controls, (p < 0.005) and Jeddah firefighters, (p < 0.0001) as shown in tables 3.10 and 3.11 respectively.

Also, of the most important liver function tests are the measurement of serum protein and protein metabolites such as urea nitrogen. The present study showed non-statistically significant differences in serum total protein, while serum albumin was found to be statistically significantly higher, (p < 0.05) in Yanbu firefighters over the normal control group.

Serum urea nitrogen measures the amount of urea nitrogen, a waste product of protein catabolism by the liver. An elevated serum urea nitrogen may be caused by impaired renal function, congestive heart failure as a result of poor renal perfusion and dehydration[16]. The present results revealed that, serum urea nitrogen was statistically significant elevated in Jeddah firefighters, (p < 0.001) and Yanbu firefighters, (p < 0.05) as compared to normal control group.

Abeloff, *et al *[17], found significant correlations between serum polycyclic biphenyls, (PCBs) concentrations and levels of liver enzymes and lipids, but mean levels of these biochemical parameters were not associated with reported exposure after adjustment for relevant covariables. Following an electrical transformer fire, serum liver functions were normal or unchanged from preexposure baselines in 60 firefighters. Such studies might support the finding presented in the present study.

Concerning kidney functions, no statistically significant differences were found among the three groups of the present study as concerns serum uric acid and creatinine. Other studies showed no significant differences were found between firefighters and normal controls, except for creatinine which decreased for both firefighters, (p < 0.001) and controls, (p < 0.01)[18].

Hyperkalemia may result from a shift of intracellular potassium into the circulation, which may occur in firefighters with the rupture of red blood cells, (hemolysis) or tissue damage, (e.g., severe burns) [19]. However, in the present work, Jeddah and Yanbu firefighters did not show any change in their serum potassium as compared to either normal control group or to each other.

One cannot evaluate total body chloride stores from the serum chloride concentration [20]. However, the present study showed that serum chloride in Yanbu firefighters was statistically significant less, (p < 0.005) as compared either to the normal control group or to Jeddah firefighters. This could be attributed to environmental and nutritional factors prevealing in Yanbu. Smith, *et al*.[21] reported that, plasma levels of sodium were elevated immediately post-firefighting and were significantly reduced below resting levels following firefighting activity. In fact, hyponatremia is a serious concern for athletes and workers who lose a great deal of sweat. Plasma volume decreases immediately following firefighting, but it returned to baseline following recovery and aggressive rehydration. sodium concentrations were significantly lower than pre-test, or immediately post-fire fighting values, after recovery[22]. The present study confirms this only in Yanbu firefighters as concerns serum sodium, (p < 0.05) and chloride, (p < 0.005).

Since serum inorganic phosphate is only a minute portion of body phosphate, alterations in the serum level can occur when the body phosphate is low, normal or high[23]. The present study represented no statistically significant differences between the studied groups as concerns serum inorganic phosphate levels.

In this study, results of lipid profile in Jeddah firefighters indicated that, only low density lipoprotein cholesterol, (LDL-C) was statistically significantly elevated, (p < 0.01) as compared to normal control group. However there was no statistically significant change in all lipid profile of Yanbu firefighters as compared to Jeddah firefighters and normal control group. Kelly, *et al*. [18] and Glueck, *et al*.[24] and established a health surveillance program for firefighters. They found that serum lipid profile was normal or unchanged from preexposure baselines. The lipid profile of firefighters did not change much from normal control group except for the Jeddah firefighter LDL-C mentioned above. The lipid profile in relation to other cardiovascular disease risk factors in 321 firefighters was evaluated at a baseline examination. The average cholesterol level in firefighters declined at the follow-up examination, (p < 0.0001). Conversely, triglycerides increased over time. The proportion of firefighters taking lipid-lowering medications increased from 3% at baseline to 12% at follow-up (p < 0.0001). Cholesterol levels declined significantly, and treatment rates for elevated cholesterol increased over time[25].

Our results indicated that, there was no statistically significant change in blood glucose level on comparison between all studied groups. The decrease in blood glucose following 90 min. of recovery is of potential concern for fire fighters. Although the recovery blood glucose value was still within a normal range, it is relatively low. In fact, approximately 30% of the fire fighters were clinically hypoglycemic at the end of the recovery period. Given that symptoms of hypoglycemia include weakness, nervousness, anxiety, and sweating, this could be a serious problem for fire fighters. The low blood glucose values suggest that following strenuous fire fighting activity a fire fighter may benefit from consuming carbohydrates, in addition to replacing fluid loss, prior to subsequent activity [21]. Firefighters had significantly increased risk for incident Diabetes Mellitus, (DM) Type-2 against clerical workers, but the significance disappeared after adjustments for BMI [26].

Cortisol measurements are used as a direct monitor of adrenal status and an indirect measure of pituitary hyper or hypo function. Elevated cortisol level is associated with adrenal tumors, pituitary tumors or ectopic ACTH-producing tumors [27]. In the present study serum cortisol level was statistically significant decreased in Yanbu firefighters as compared to normal control group. However, there was no statistically significant change in Jeddah firefighters as compared to normal control group and Yanbu firefighters, in contradiction with the other following two studies: the first study reported that over 1 year, 72 male firefighters completed the Daily Stress Inventories, for 2 shift cycles, (16 days), every 3 months. In contrast to expectations, as daily stress decreased across the year, salivary cortisol increased and testosterone levels decreased. Within-subjects comparisons of the sessions with the highest and lowest stress confirmed these linear relationships: Lower stress prior to the assessment session was associated with higher cortisol levels [28]. At the same time plasma levels of ACTH and cortisol were significantly elevated post firefighting activity and cortisol remained elevated following 90 min. of recovery. Elevated cortisol immediately following activity was related to reduced feelings of energy. These data demonstrate the magnitude of the physiological and psychological disruption following strenuous firefighting activity [21].

Any elevated CK result is automatically reflexes to a myocardial infarction and muscle diseases. Creatine kinase may also be elevated following muscle injury or strenuous exercise [29]. In this study, CK was statistically significantly increased in Jeddah firefighters, (p < 0.005) and Yanbu firefighters, (p < 0.05) as compared to normal control group. However, there was no statistically significant difference between Yanbu firefighters and Jeddah firefighters as shown in table. In a single case study, Ottervanger, *et al*. [30] reported that creatine kinase level raised to a maximum of 3,277 U/L (normal, < 100 U/L) in a 39 years old cigarette smoking fireman.

Lactate dehydrogenase is most often measured to evaluate the presence of tissue or cell damage[16]. In the present study, lactate dehydrogenase was statistically significantly elevated in Jeddah firefighters, (p < 0.005) and Yanbu firefighters, (p < 0.05) as compared to normal control group, while was less in Yanbu firefighters, (p < 0.01) as compared to Jeddah firefighters. Penney and Maziarka, 1976 found that, there was a significant elevation in LDH activity post exposure to fire smoke in firefighters.

## Conclusion

Such results might point out to the need for more health protective and prophylactic measures to try to avoid such hazardous health effects that might endanger firefighters under their highly drastic working conditions. Besides using of personal protective equipments for firefighters to protect them against exposure to toxic materials of fire smoke, it is recommended that, firefighters must be under continuous medical follow up through a standard timetabled medical laboratory investigations to allow for early detection of any biochemical or hematological changes that might happen during their service lives and to allow for early treatment whenever necessary

## Competing interests

The authors declare that they have no competing interests.

## Authors' contributions

(AA) planning and design the protocol, carried out the experiments and drafted the manuscript. (AR) performed the statistics, analysis the results and comments the discussions. (MA) participated in its design, experiments design, collection samples and coordination. All authors read and approved the final manuscript.
